# Trends in Subjective Quality of Life Among Patients With First Episode Psychosis—A 1 Year Longitudinal Study

**DOI:** 10.3389/fpsyt.2019.00053

**Published:** 2019-02-13

**Authors:** Xiao Wei Tan, Shazana Shahwan, Pratika Satghare, Boon Yiang Chua, Swapna Verma, Charmaine Tang, Siow Ann Chong, Mythily Subramaniam

**Affiliations:** ^1^Research Division, Institute of Mental Health, Singapore, Singapore; ^2^Early Psychosis Intervention Program (EPIP), Institute of Mental Health, Singapore, Singapore; ^3^Duke-NUS Medical School, Singapore, Singapore; ^4^Lee Kong Chian School of Medicine, Nanyang Technological University, Singapore, Singapore

**Keywords:** first episode psychosis, subjective Quality of Life, educational achievement, employment, longitudinal study

## Abstract

Quality of life (QoL) is often used as an outcome assessment in programs treating patients with first-episode psychosis (FEP). The aim of this study was to examine the longitudinal trend of subjective QoL among patients with FEP and identify the potential influence of patients' social-demographic/lifestyle factors on the trend of QoL. Two hundred and eighty subjects participated in the study. Patient's demographics and subjective QoL were collected at baseline, 6 months and 1 year follow-up. Data were analyzed with a fixed-effect general linear regression model. Subjective QoL demonstrated significant trends of improvement in all four subdomains (physical health, psychological health, social relationships, and environment). Compared with unemployed participants, employed participants were significantly associated with better social relationships (*p* = 0.005) and environment (*p* = 0.029) after adjusting for age and gender. Moderation analysis demonstrated a significant improvement of physical health, social relationships, and environment for participants with a higher level of educational achievement, but not for participants with a lower level of educational achievement. Our results indicate that patients with FEP experienced significant improvement in subjective QoL over a 1 year period. Being employed was associated with overall better social relationships and environment among patients with FEP and higher educational achievement was associated with improvement of physical health, social relationship, and environment. Hence, educational achievement and employment could be considered for future optimization of early psychosis intervention programs.

## Introduction

The World Health Organization (WHO) has defined QoL as individuals' perception of their position in life in the context of the culture and value systems in which they live and in relation to their goals, expectations, standards, and concerns (1). In accordance with the definition of health by WHO, subjective Quality of Life (QoL) covers physical, emotional, mental, social, and behavioral components of well-being and function as perceived by each individual (2). Subjective QoL was recommended as a valuable outcome assessment in programs treating patients with schizophrenia (3, 4), and patients with first episode psychosis (FEP) (5). Early treatment of patients with psychotic symptoms can result in a significant reduction of morbidity, suicide rate, improved subjective QoL, and functional recovery (5–9). Although studies have suggested a significant improvement in objective QoL over the 1st year in the treatment of patients with FEP (10), there is still a lack of conclusive evidence concerning the course of subjective QoL. Few existing studies suggest that subjective QoL does not appear to improve over time and that it remains stable both during short (11) and long periods of follow-up (12), while other studies demonstrated an improvement of subjective QoL over the years of follow-up (13, 14). Moreover, among patients with FEP, the associations between clinical characteristics, such as psychotic and cognitive symptoms, and subjective QoL have been inconsistently reported. Symptomatic remitters of positive and psychotic symptoms were reported to be associated with higher levels of subjective life satisfaction and functioning (15, 16). Severe positive and negative symptoms were strongly related to poor QoL among outpatients with schizophrenia (13, 17), while QoL was also reported to be correlated with both psychotic and negative symptoms to a minor extent (18). These inconclusive findings were most probably due to the heterogeneity of study design, patient setting, methods of recruitment, premorbid adjustment, varying instruments that were used for assessment of QoL and different approaches of statistical analysis.

The factors influencing QoL of patients with FEP remains unclear. Higher depressive symptoms and lower daily activities had a negative effect on subjective QoL and this independent effect diminished over time (13). Educational achievement in patients with chronic schizophrenia was reported to be either positively (19) or negatively (20) associated with subjective QoL, which was influenced by the individual's cognitive difficulties, personal adaptive skills, resilience as well as environmental-social factors and support. For patients with psychiatric disabilities, employment plays an essential role in providing financial gains, social contacts, and support, as well as a sense of personal achievement (21). Being employed was associated with better health related QoL for patients with chronic schizophrenia (22–24). Results from the NAVIGATE study indicated that, compared with usual community care, comprehensive care improved the subjective QoL, and psychopathology among patients with FEP (25). Secondary analysis of data from the NAVIGATE study showed that a program with supported employment and education (SSE) was associated with improvement in work or school participation among patients with FEP (26). However, the influence of SSE on participants' subjective QoL remains unclear.

## Purpose of Study

The primary goal of the current study was to examine the trend of subjective QoL among patients with FEP over 1 year of treatment in the early psychosis intervention program (EPIP). We further aimed to identify the potential association of significant confounders including educational achievement and employment, with the trend of subjective QoL.

## Methods

### Sample

This single center cohort study enrolled outpatients with FEP diagnosed at the Institute of Mental Health, Singapore, which is the de facto national mental health institute of the country and a tertiary treatment center that serves the entire population of Singapore. FEP was defined as the first episode of psychotic disorder with no prior or minimal treatment (<12 weeks of antipsychotic medication) (27). The recruitment for the current study started in Feburary 2014 and ended in October 2016, with the last follow-up conducted on October 2017. The inclusion criteria for the participants were: (i) aged between 16 and 40 years and (ii) no history of major medical or neurological illness. Ethical approval to conduct the study was obtained from the National Healthcare Group Domain Specific Review Board. All participants provided written informed consent. Parental consent was obtained for participants who were below the age of 21 years. The EPIP in Singapore was implemented to provide universal and indicated prevention for patients with FEP, with the primary goals of improving clinical outcomes and QoL, as well as reducing the cost and burden of care for their families and the general public. The program comprises several initiatives. (1). Education of the general public with the major goal of reducing the duration of untreated psychosis (DUP). (2). Networking with primary healthcare providers. (3). Providing decentralized and accessible services. (4). Tertiary prevention aimed at reducing mortality, morbidity and the progression of the illness, provided by a multidisciplinary team (psychiatrists, case managers, psychologists, social workers, occupational therapists, pharmacologists, and nurses). The details of the EPIP in Singapore have been described in previous articles (27, 28).

### Measures

Baseline assessment included data on participants' social demographics and clinical history. Severity of symptoms was assessed using the Positive and Negative Syndrome Scale (PANSS) for schizophrenia (29) while functioning was assessed with the Global Assessment of Functioning (GAF) score (30). These ratings were performed by psychiatrists who were trained in the use of the rating instruments (9). The inter-rater reliability for PANSS in our sample was 0.94. PANSS, GAF score, prescription of antipsychotics, antidepressants, and mood stabilizers were collected from medical records.

Hazardous alcohol use was estimated using the Alcohol Use Disorders Identification Test (AUDIT, self report version), which is a brief, 10 item inventory developed by the World Health Organization (WHO). Responses to the ten AUDIT questions were assigned a score between 0 and 4, based on the frequency of the circumstance or activity described. Scores of 8 or higher suggest a possibility of hazardous alcohol use, and a need for further monitoring or assessment (31). AUDIT has been used among patients with FEP in Singapore to measure hazardous alcohol use (32).

The WHOQOL-BREF is a 26 item questionnaire that is designed to measure an individual's perception of QoL over the past 1 month (33). The WHOQOL-BREF consists of 4 domains: physical health, psychological health, social relationships, and environment. All items are constructed on variations of a 5-point Likert scale, with scores from 1 to 5, enquiring on “how much,” “how completely,” “how often,” “how good,” or “how satisfied” the individual felt. Scores for the 4 domains were calculated by taking the mean of all items within the domain and multiplying by 4 and transforming it to a 0–20 scale. Domain scores were not scored when more than 20% of the items were missing. It was also not calculated when more than 2 items were missing from the domain. This is, with the exception of domain 3 (social relationship), where it is unacceptable if one item is missing (34). This instrument has been validated in patients with schizophrenia, reporting good internal consistency for total WHOQOL-BREF score and being adequate for the 4 domains (35). In our current study, QoL of participants was assessed at baseline, 6 months and 1 year follow-up.

For statistical analysis, patient characteristics were regrouped. Educational achievement, “Low” included those with General Certificate of Education (GCE) “O” level (or equivalent) and lower qualifications; “High” included those with higher than GCE “O” level qualifications. Participant's employment status was self-reported by answering the question “What was your main working status over the past 1 year.” Participants with the answer “full-time/part-time employment,” “on national service,” and “student” were grouped as “Employed.” Those who answered, “home maker/house wife” or “jobless” were grouped as “Unemployed.” “Unmarried” referred to participants who were never married, separated, divorced or widowed. “Married” referred to participants who were currently married. “Housing type” was defined as the current housing condition regardless of whether it was self-owned or rented. “Economic house” referred to all government developed housing and “Private house” referred to all private housing developments including condominium, terrace houses and bungalows. Baseline data on smoking was collected by asking participants if they were smokers with the additional options of “ex-smoker,” “never smoked,” or “currently smoking.” Participants who answered “ex-smoker” and “never smoked” were grouped as “non-smokers.” Participants who answered that they were “currently smoking” were grouped as “current smokers.”

Two hundred and eighty patients were consecutively enrolled in this study and 81 of them completed the assessments at all three time points.

### Data Analysis

All statistical analyses were performed with SPSS (*IBM, v.25*). We used descriptive statistics to establish the socio-demographic and clinical characteristics of the study cohort. Numerical variables were presented as mean ± standard deviation (SD) for variables with normal distribution and median (interquartile range, IQR) for variables with skewed distribution. Categorical variables were presented as count (percentage, %). Comparison analysis between the participants who presented and those who were lost to follow-up at either the 6 months or 1 year visit were performed with *t*-test, chi-square test, or Mann-Whitney *U*-tests to determine the differences in socio-demographic and clinical characteristics. The actual mean scores of QoL collected at baseline, 6 months follow-up and 1 year follow-up were compared with Analysis of Variance (ANOVA) with a *post-hoc Bonferroni* test and the actual mean score of PANSS or GAF scale collected at baseline and 1 year follow-up were compared with a paired *t*-test.

The association of participant educational achievement and employment status with the course of QoL was analyzed by the fixed-effect linear mixed regression model (LMM). LMM with repeated measurement was used to estimate the within-subject trend of QoL, PANSS score, GAF score, and the moderation (interaction) between participant's social demographics and the course of QoL. In the mixed regression model, QoL score was treated as a dependent variable. Patients' characteristics and index for repeated measurement were treated as independent variables. Interaction terms which were built between social-demographic/lifestyle factors and index of repeated measurement were included into the adjusted LMM model, providing the *p*-value for the interaction terms were <0.05 before adjustment. The interaction term between educational achievement and the trend of QoL was included in the final model as the interaction was statistically significant. Mean values of subgroup QoL score at various time points were estimated by treating the index of repeated measurements as a categorical variable in the regression model and the estimated mean scores were exported into an Excel document for plotting. The repeated covariance type for LMM was set at AR(1) to achieve lowest value of Akaike information criterion (AIC) and Bayesian information criterion (BIC). Statistical significance was accepted at the ≤ 0.05 level for all tests.

## Results

Of the 280 patients who were included in the study, 136 completed the 6 months follow-up and 129 completed the 1 year follow-up. Participants' baseline demographics and clinical characteristics are shown in Table 1. 91.2% of the participants were patients diagnosed with schizophrenia and related psychosis and 8.2% were patients diagnosed with mood disorder with psychotic symptoms. At 6 months, Chinese patients (79.4%) were more likely to continue with the study follow-up compared with patients in other ethnic groups (*p* = 0.024, Table 1). Patients who were Singaporeans (97.8%) were more likely to continue with the study follow-up compared with foreigners (*p* = 0.015). At 1 year, unmarried patients (92.2%) were more likely to participate in the study compared with married participants (*p* = 0.038).

**Table 1:** Comparison of patient characteristics of those with and without 6 months and 1 year follow up.

**Patient characteristics**	**Baseline (*n* = 280)**	**6M with follow up (*n* = 136)**	**6M without follow up (*n* = 144)**	***p*-value**	**1 year with follow up (*n* = 151)**	**1 year without follow up (*n* = 129)**	***p*-value**
Age, years, mean ± SD	25.76 ± 6.23	25.24 ± 5.80	26.26 ± 6.61	0.174	25.03 ± 5.97	26.39 ± 6.41	0.067
Sex, no. (%)				0.234			0.561
Male	142 (50.7)	64 (47.1)	78 (54.2)		79 (52.3)	63 (48.8)	
Female	138 (49.3)	72 (52.9)	66 (45.8)		72 (47.7)	66 (51.2)	
Ethnicity, no. (%)				0.024^a^			0.227
Chinese	200 (71.4)	108 (79.4)	92 (63.9)		107 (70.9)	93 (72.1)	
Malay	41 (14.6)	13 (9.6)	28 (19.4)		19 (12.6)	22 (17.1)	
Indian	25 (8.9)	11 (8.1)	14 (9.7)		18 (11.9)	7 (5.4)	
Others	14 (5.0)	4 (2.9)	10 (6.9)		7 (4.6)	7 (5.4)	
Nationality, no. (%)				0.015^a^			0.123
Singaporean	262 (93.6)	133 (97.8)	129 (89.6)		138 (91.4)	124 (96.1)	
Permanent resident	14 (5.0)	3 (2.2)	11 (7.6)		9 (6.0)	5 (3.9)	
Others	4 (1.4)	0 (0.0)	4 (2.8)		4 (2.6)	0 (0.0)	
Marital Status, no. (%)				0.851			0.038^a^
No	246 (87.9)	120 (88.2)	126 (87.5)		127 (84.1)	119 (92.2)	
Yes	34 (12.1)	16 (11.8)	18 (12.5)		24 (15.9)	10 (7.8)	
Children, no. (%)				0.392			0.162
Without children	253 (90.4)	125 (91.9)	128 (88.9)		133 (88.1)	120 (93.0)	
With children	27 (9.6)	11 (8.1)	16 (11.1)		18 (11.9)	9 (7.0)	
Educational achievement, no. (%)				0.915			0.224
Low	77 (27.5)	37 (27.2)	40 (27.8)		37 (24.5)	40 (31.0)	
High	203 (72.5)	99 (72.8)	104 (72.2)		114 (75.5)	89 (69.0)	
Father education, no. (%)				0.259			0.557
Low	197 (70.4)	97 (67.4)	100 (73.5)		47 (31.1)	36 (27.9)	
High	83 (29.6)	47 (32.6)	36 (26.5)		104 (68.9)	93 (72.1)	
Mother education, no. (%)				0.882			0.321
Low	207 (73.9)	100 (74.3)	107 (74.3)		108 (71.5)	99 (76.7)	
High	73 (26.1)	36 (26.5)	37 (25.7)		43 (28.5)	30 (23.3)	
House own^b^, no. (%)				0.756			0.395
Private	26 (10)	12 (9.4)	14 (10.5)		16 (11.4)	10 (8.3)	
Economic	235 (90)	116 (90.6)	119 (89.5)		124 (88.6)	111 (91.7)	
Employment status^b^, no. (%)				0.906			0.378
Unemployed	181 (66.1)	87 (66.4)	94 (65.7)		93 (63.7)	88 (68.8)	
Employed	93 (33.9)	44 (33.6)	49 (34.3)		53 (36.3)	40 (31.3)	
Smoking status, no. (%)				0.621			0.114
Ex or never smoker	168 (60.0)	83 (61.0)	92 (63.9)		88 (58.3)	87 (67.4)	
Current smoker	112 (40.0)	53 (39.0)	52 (36.1)		63 (41.7)	42 (32.6)	
Alcohol, no. (%)				0.369			0.570
No hazardous use	244 (87.1)	116 (85.3)	128 (88.9)		130 (86.1)	114 (88.4)	
With hazardous use	36 (12.9)	20 (14.7)	16 (11.1)		21 (13.9)	15 (11.6)	
Diagnosis,^b^ no. (%)							
Schizophrenia and related Psychosis	212 (75.7)	113 (83.1)	99 (68.8)	0.489	116 (63.6)	96 (74.4)	0.078
Mood disorder with Psychotic symptoms	23 (8.2)	14 (10.3)	9 (6.2)		17 (11.3)	6 (4.7)	
PANSS_P^b^, mean ± SD	21.89 ± 5.99	22.11 ± 6.12	21.71 ± 5.91	0.613	22.27 ± 6.19	21.60 ± 5.85	0.400
PANSS_N^b^, mean ± SD	15.76 ± 8.73	15.72 ± 8.60	15.78 ± 8.88	0.957	16.32 ± 8.61	15.32 ± 8.84	0.382
PANSS_GPS^b^, mean ± SD	38.16 ± 11.35	38.42 ± 10.48	37.94 ± 12.06	0.744	39.35 ± 10.74	37.24 ± 11.75	0.151
GAF_S^b^, mean ± SD	44.57 ± 12.21	43.50 ± 12.15	45.47 ± 12.23	0.218	43.56 ± 12.25	45.34 ± 12.17	0.267
GAF_D^b^, mean ± SD	46.57 ± 11.60	45.58 ± 11.39	47.39 ± 11.75	0.232	46.15 ± 11.82	46.89 ± 11.46	0.628
No. of antipsychotics, median (IQR)	1 (0)	1 (0)	1 (0)	0.777	1 (0)	1 (0)	0.880
No. of antidepressants, median (IQR)	0 (1)	0 (1)	0 (1)	0.651	0 (1)	0 (1)	0.692
No. of mood stabilizers, median (IQR)	0 (0)	0 (0)	0 (0)	0.097	0 (0)	0 (0)	0.175
DUP^b^, days, mean ± SD	13.55 ± 21.69	13.28 ± 20.37	13.79 ± 22.85	0.856	12.41 ± 20.60	14.43 ± 22.53	0.475

a *p < 0.05*.

b *Data may not sum to total due to missing values*.

*SD, Standard deviation; IQR, Interquartile range; PANSS_P, Positive and negative syndrome scale_ positive; PANSS_N, Positive and negative syndrome scale _negative; PANSS_GPS, Positive and negative syndrome scale _general psychopathology scale; GAF_S, Global assessment of functioning_symptoms; GAF_D, Global assessment of functioning_disabilities; DUP, Duration of untreated psychosis*.

**Figure 1:**
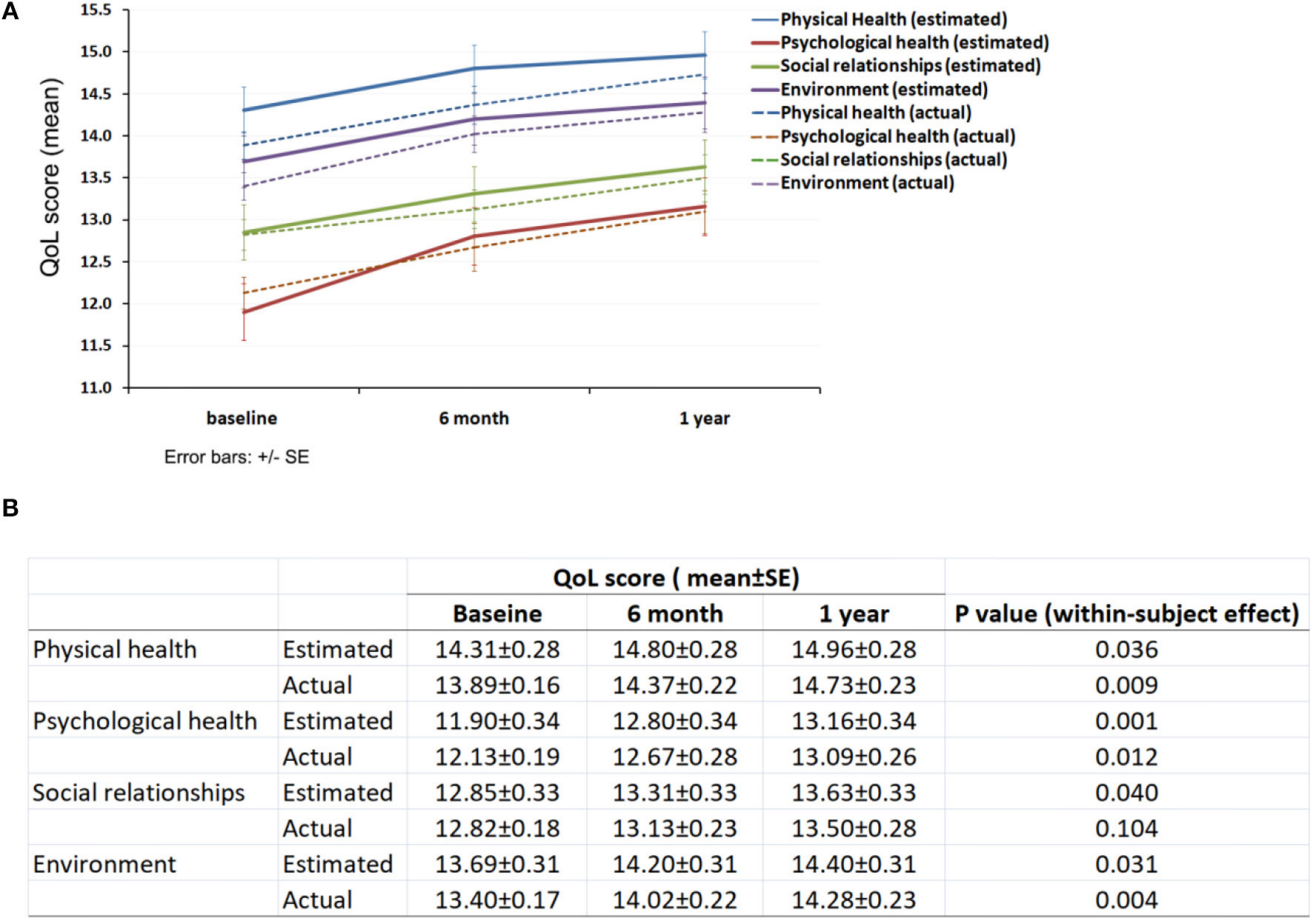
QoL of the participants. **(A)** Trends of subjective QoL over 1 year follow-up period; **(B)** Subdomain QoL score at baseline, 6 months and 1 year.

Participants reported improved QoL in all four subdomains over the 1 year period (Figure 1A). In domain 1 (physical health), the estimated mean score of QoL improved from 14.31 ± 0.28 to 14.96 ± 0.28 (Figure 1B). Overall *p*-value for this trend of QoL was 0.036. In domain 2 (psychological health), the estimated mean score of QoL significantly increased from 11.9 ± 0.34 to 13.16 ± 0.34 (*p* < 0.001). In domain 3 (social relationships), the estimated mean score of QoL improved from 12.85 ± 0.33 to 13.63 ± 0.33, with an overall *p*-value of 0.04. In domain 4 (environment), the estimated mean score of QoL improved from 13.69 ± 0.31 to 14.4 ± 0.31. *P*-value for the trend of QoL was 0.031. The actual mean score of QoL showed similar trends to the estimated mean score with *p* < 0.05 for subdomains of physical health, psychological health and environment.

**Figure 2:**
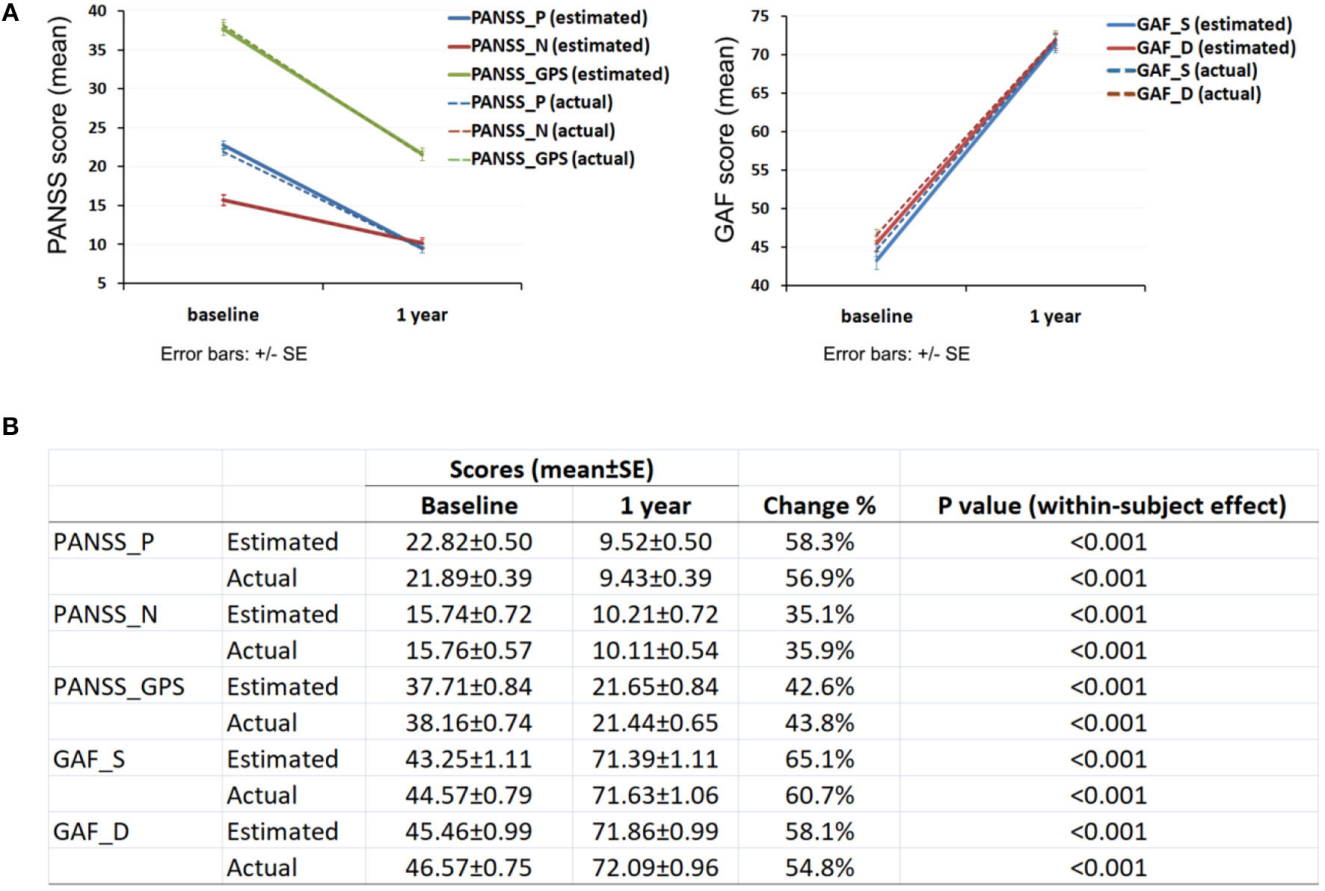
Clinical assessment of participant's psychotic symptoms and function. **(A)** Change of PANSS and GAF score over 1 year; **(B)** PANSS and GAF score at baseline and 1 year. PANSS_P, Positive and negative syndrome scale_ positive; PANSS_N, Positive and negative syndrome scale_negative; PANSS_GPS, Positive and negative syndrome scale_general psychopathology scale; GAF_S, Global assessment of functioning_symptoms; GAF_D, Global assessment of functioning_disabilities.

Clinical assessments demonstrated an overall reduction in psychotic symptoms and improvement in function as indexed by PANSS and GAF scores, respectively (Figure 2A). PANSS score decreased by about 58.3% for positive symptoms (within-subject *p* < 0.001, Figure 2B); 35.1% for negative symptoms (*p* < 0.001) and 42.6% for general psychopathology (*p* < 0.001) over the 1 year follow up. GAF score increased by about 65.1% for the assessment of symptoms (*p* < 0.001) and increased by about 58.1% for the assessment of disabilities (*p* < 0.001). Actual mean score of PANSS and GAF showed similar trends to the estimated score with *p* < 0.001 for all subcategories.

Regression analysis showed no significant association between participant's educational achievement and overall QoL both before and after adjustment (Table 2). Compared with patients who were unemployed, patients who were employed were associated with better social relationships [adjusted B: 1.73, 95% CI: 0.55–2.93, *p* = 0.005] and environment [adjusted B: 1.29, 95% CI: 0.13–2.44, *p* = 0.029) (Table 2).

**Table 2:** Association of participant's educational achievement or employment status with overall subjective QoL during follow-up period.

**Variable**	**Outcome**		**Before adjustment**				**After adjustment**^**a**^		
		**95% CI**				**95% CI**			
		**B**	**Lower bound**	**Upper bound**	***p*-value**	**B**	**Lower bound**	**Upper bound**	***p*-value**
Education (high vs. low)	Physical health	0.13	−0.93	1.18	0.813	0.44	−0.65	1.52	0.427
	Psychological health	−0.68	−1.96	0.61	0.297	−0.50	−1.83	0.84	0.461
	Social relationships	0.04	−1.18	1.25	0.952	0.43	−0.81	1.68	0.491
	Environment	0.35	−0.82	1.53	0.552	0.89	−0.27	2.05	1.131
Employment (employed vs. unemployed)	Physical health	0.51	−0.47	1.48	0.305	0.32	−0.76	1.40	0.555
	Psychological health	0.52	−0.71	1.76	0.404	0.64	−0.70	1.99	0.344
	Social relationships	1.74	0.66	2.82	0.002	1.73	0.55	2.93	0.005
	Environment	1.41	0.33	2.49	0.011	1.29	0.13	2.44	0.029

a *Adjusted for age and gender*.

*CI, Confidence interval; B, Beta coefficient*.

**Figure 3:**
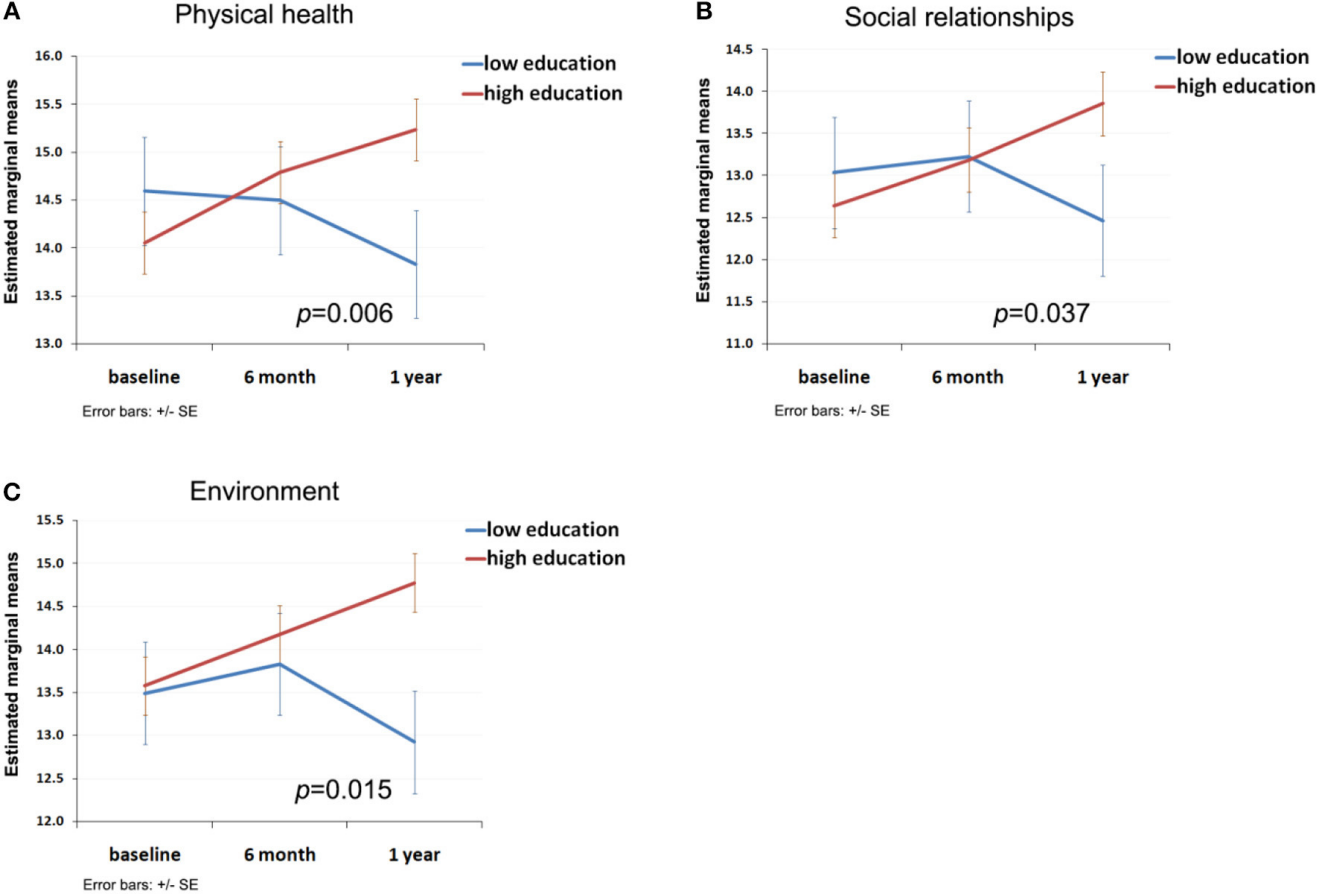
Moderation effect of participant's educational achievement on the trend of subjective QoL. **(A)** Moderation curve of educational achievement with trend of physical health; **(B)** Moderation curve of educational achievement with trend of social relationships; **(C)** Moderation curve of educational achievement with trend of environment.

Moderation analysis identified a continuous and significant improvement of physical health (domain 1, Figure 3A), social relationships (domain 3, Figure 3B) and environment (domain 4, Figure 3C) over a 1 year period for participants with higher level of educational achievement, but not for participants with a lower level of educational achievement (*p* = 0.006, 0.037 and 0.015, respectively). The moderation relationship between educational achievement and psychological health was borderline (*p* = 0.09) and there was no significant moderation relationship between employment status and the four subdomains of QoL.

## Discussion

In the present study, patients with FEP demonstrated a significant improvement of psychotic symptoms, general functioning and subjective QoL during the 1 year follow-up period. To our best knowledge, this study is among the first few studies that have examined the temporal development of subjective QoL among patients with FEP using a model of repeated measurements with multiple time points of assessment.

Although there have been many studies on the determinants of QoL among patients with mental disorders (36, 37), there is a lack of consensus as to how QoL should be defined and measured. For patients with schizophrenia, the validity of subjective QoL might be limited by a self-reporting scale (2) and could have been influenced by several factors including patients' persistent psychotic symptoms, self-esteem, adaptation to adverse circumstances (38), presence of cognitive deficits and lack of insight (20, 36). However, some studies have demonstrated the convergent validity of QoL assessed by a patient's self-report and that assessed objectively by clinicians (39). Patients with schizophrenia were able to report their feelings, experiences, and social functions accurately (37, 40, 41), showing that the QoL of patients with psychosis can be assessed subjectively.

Our study allows the testing of potential factors influencing the trend of QoL over time. The literature on the relationship between QoL and education in schizopherenia is inconclusive. In some studies, patients with a higher level of education reported worse QoL compared to patients with lower levels of education (20). While, others demonstrated that in patients with schizophrenia, higher educational achievement was correlated with better social functioning and greater satistifaction with life (42). In the current study, we found no evidence of significant association between participants' educational level and overall QoL. However, compared to participants with lower educational achievement, participants with higher educational achievement were more likely to report worse physical health and social relationships at baseline, which is possibly due to the higher social demands and expectations among this group of patients.

The results of the associations between employment and overall QoL in this study appear to be consistent with previous studies in the literature. Patients who were employed were likely to be associated with better health-related QoL compared with patients who were unemployed (43). The association may be explained by the better self-esteem among patients with employment, which was described as a mediating factor between being employed and QoL (44), and having a larger social network due to being employed (45). The causal relationship between employment and QoL remains unclear and it is possible that the participants with higher QoL were more likely to be employed.

During the 1 year follow-up period, compared to patients with a relatively lower level of educational achievement, patients with a higher level of educational achievement demonstrated a continuous and significant improvement of QoL in almost all four subdomains (physical health, social relationships, environment support, and psychological health). This pattern was similarly reported in a previous study which showed that graduates were more resilient in the face of adversities, and stressful circumstances such as divorce and ill-health as compared to non-graduates (46). Across three measures of well-being—life satisfaction, happiness and worthwhileness—graduates reported greater well-being even when confronting challenging life events, although graduates tended to be more anxious than non-graduates when in good health. Hence, it is reasonable for us to speculate that higher level of education may benefit patients with mental health disorders, especially when confronting episodes of psychosis. Indeed, students with successful post-secondary level of school education have been found to be able to continuously develop coping strategies to overcome cognitive difficulties while they are suffering from early episodes of psychosis (47).

We observed no moderation effect of employment status with the trend of QoL although being employed was associated with, overall, better social relationships and environment. Compared with patients who were unemployed, patients who were employed may have better financial resources to support the treatment, respond better to the treatment with regards to medication/therapy adherence, and enjoy better co-operation with their primary attendants and other care-givers. Participants being employed may have better social relationships or resources to start with, and these resources in turn may help them to have a better QoL at both baseline and during the follow-up period.

It has been reported that there is higher school dropout (15, 48) as well as unemployment (49–52) among young adults with schizophrenia after the first episode of their illness than in the general population. Young adults with FEP have been observed to frequently disregard the suggestions from service providers and fail to return to school (53). Many young patients develop psychosis which can interfere with their ability to fulfill their occupational goals. Findings from the Singapore Mental Health Study in 2010 revealed that the rate of unemployment among those with common mental illnesses was 11.1% which was significantly higher than the 6.7% rate of unemployment in those without mental illness. The data also showed that the rate of mental illness in people who were unemployed was twice as high as compared to those who were employed (5.3 vs. 2.3%) (54). Singapore has a multi-racial culture, influenced by South Asian, East Asian, and Eurasian cultures. Singapore has a high standard of living and low unemployment rates. Meritocracy is valued in Singapore and this results in promoting competitiveness for job and prestige in the society (55, 56). Employment and education are therefore highly valued in Singapore and being employed may thus contribute fundamentally to their QoL. Our study emphasizes the influential role of education and employment on the subjective QoL among patients with FEP.

We observed a significant amelioration of overall positive symptoms, negative symptoms and general pathological symptoms as well as a significant improvement of general functioning among our participants over the follow-up period which was possibly caused by the early treatment of psychosis or reasons that we didn't explore in this study. We observed no association of participants' educational achievement and employment status with the overall change of psychiatric symptoms and clinical assessment of general functioning. Nor did we find any interaction relationship between participants' educational achievement and employment status and the change of psychiatric symptoms and general functioning. Hence for patients with FEP, the association and moderation role of patients' educational achievement and employment status on the severity of clinical symptoms and clinical assessment of general functioning was not identified in our model. We were not able to conduct a trend analysis of changes in PANSS and GAF scores in this study as data was available for only two time points (baseline and 1 year). We analyzed the association between the 1 year change of PANSS and GAF score with the change of QoL using generalized linear regression. No significant associations were observed which was in line with previous findings that after comprehensive treatment, subjective QoL among patients with FEP was correlated with both psychotic and negative symptoms, but only to a minor extent (16). Future studies should consider incorporating measures of both socio-demographic and clinical correlates (e.g., medications and psychotherapy) over time to conduct a more robust trend analysis of QoL both in patients with FEP as well as other illnesses to ensure a better understanding of modifiable factors.

In summary, the main strength of our study is the repeated measurements at multiple time points that were used to examine the trend of QoL among those with FEP. The local setting, self-reporting nature of study involving patients with psychosis, and potential bias due to the selective loss of follow-up may limit the generalization of current findings to a global population. We have identified the positive association of employment status with QoL and the moderation effects of educational achievement on the trend of QoL, which could also have been pre-conditioned by other confounders that we didn't explore in this study. Although this secondary analysis should be interpreted cautiously and considered exploratory, our study suggests that it is important for patients with FEP to have age appropriate roles i.e., they return to school or employment as early as possible.

## Author Contributions

XT, SV, CT, SC, and MS contributed conception and design of the study. SS, PS, and BC contributed to data collection. XT and MS organized the database and wrote the first draft of the manuscript. All authors contributed manuscript revision, and approved the submitted version.

### Conflict of Interest Statement

The authors declare that the research was conducted in the absence of any commercial or financial relationships that could be construed as a potential conflict of interest.
